# Porcine Noroviruses Related to Human Noroviruses

**DOI:** 10.3201/eid1112.050485

**Published:** 2005-12

**Authors:** Qiu-Hong Wang, Myung Guk Han, Sonia Cheetham, Menira Souza, Julie A. Funk, Linda J. Saif

**Affiliations:** * The Ohio State University, Wooster, Ohio, USA; † The Ohio State University, Columbus, Ohio, USA

**Keywords:** norovirus, calicivirus, porcine, recombinant, research

## Abstract

Detection of genogroup II (GII) norovirus (NoV) RNA from adult pigs in Japan and Europe and GII NoV antibodies in US swine raises public health concerns about zoonotic transmission of porcine NoVs to humans, although no NoVs have been detected in US swine. To detect porcine NoVs and to investigate their genetic diversity and relatedness to human NoVs, 275 fecal samples from normal US adult swine were screened by reverse transcription–polymerase chain reaction with calicivirus universal primers. Six samples were positive for NoV. Based on sequence analysis of 3 kb on the 3´ end of 5 porcine NoVs, 3 genotypes in GII and a potential recombinant were identified. One genotype of porcine NoVs was genetically and antigenically related to human NoVs and replicated in gnotobiotic pigs. These results raise concerns of whether subclinically infected adult swine may be reservoirs of new human NoVs or if porcine/human GII recombinants could emerge.

## Abstract 2

Pigs may be reservoirs for human noroviruses, and porcine/human genogroup II recombinants could emerge.

Noroviruses (NoVs) (family *Caliciviridae*, genus *Norovirus*) cause diarrhea in humans and animals (*1**–**3*). The NoV genome is 7.3–7.7 kb long with 3 open reading frames (ORFs) encoding a polyprotein that undergoes protease processing to produce several nonstructural proteins, including an RNA-dependent RNA polymerase (RdRp), a major capsid protein (VP1, capsid), and a minor capsid protein (VP2) (*1**,**4**,**5*). The capsid protein contains a conserved shell (S) and hypervariable protruding (P) domains (*6*). Noroviruses are genetically diverse and make up 27 genotypes within 5 genogroups, GI/1–8, GII/1–17, GIII/1–2, GIV, and GV, based on the capsid genes of 164 strains (*7*). Human NoVs cause an estimated 23 million cases of illness annually in the United States (*8*) and >90% of nonbacterial epidemic gastroenteritis worldwide (1). The low infectious dose, environmental resistance, strain diversity, shedding from asymptomatic persons, and varied transmission vehicles render human NoVs highly contagious.

Norovirus RNA was detected by reverse transcription–polymerase chain reaction (RT-PCR) in 4 of 1,017 normal slaughtered pigs in Japan (*9*) and in 2 of 100 pooled pig fecal samples in the Netherlands (*10*). These porcine NoVs (Sw43/97/JP, Sw918/97/JP, and 34/98/NET) are genetically similar and are classified into GII (*9**,**10*), like most epidemic human NoVs (*11**–**13*). Also, the viruslike particles (VLPs) of Sw918 strain cross-react with antibodies against human GII but not GI NoVs (*14*). The close genetic and antigenic relationships between human and porcine NoVs raise public health concerns regarding their potential for zoonotic transmission and as reservoirs for emergence of new epidemic human strains.

Farkas et al. (*14*) reported that US swine sera react with Po/NoV/GII/Sw918 strain, but no direct detection of NoV from US swine has been reported. To detect porcine NoVs and assess their genetic diversity and relatedness to human NoVs, we screened 275 pig fecal samples from US swine by RT-PCR with a calicivirus universal primer pair p290/110 targeting the RdRp region (*15**,**16*), followed by sequencing the 3 kb on the 3´ end of the genome for 5 NoV strains. Gnotobiotic pigs were inoculated with porcine NoVs to examine their infectivity and to produce convalescent-phase antiserum for antigenic analysis.

## Materials and Methods

Fecal samples (N = 275) were collected from December 2002 to June 2003 from finisher (10–24 weeks of age) pigs and gestating sows (>1 year of age) from 3 Ohio swine farms (10, 60, and 32 samples), 1 Ohio slaughterhouse (83 samples), 1 Michigan swine farm (61 samples), and 2 North Carolina swine farms (8 and 21 samples). Fresh fecal samples were collected from individual pigs, placed into sterile containers, and stored frozen.

Sample RNA was extracted from 10% to 20% of fecal suspensions in sterile Eagle minimal essential medium (EMEM, Invitrogen, Carlsbad, CA, USA) by using Trizol LS (Invitrogen). For some samples, RNA was concentrated and purified by using QIAamp Viral RNA Mini kit (Qiagen, Valencia, CA, USA).

RT-PCR was performed separately by using primer pair p290 (5´-GATTACTCCAAGTGGGACTCCAC-3´) (15) and p110 (5´-ACDATYTCATCATCACCATA-3´) (*16*) as previously described (*15*) but at 48°C for annealing (317 bp for NoV or 329 bp for sapovirus). To amplify the 3-kb 3´ end fragment, cDNA was synthesized by SuperScript III First-Strand cDNA synthesis kit (Invitrogen) with primer VN_3_T_20_ (5´-GAGTGACCGCGGCCGCT_20_-3´). PCR was then performed with TaKaRa Ex Taq polymerase (TaKaRa Mirus Bio, Madison, WI, USA) with primers p290 and VN_3_T_20_. Quantitative (endpoint titration) RT-PCR (*17*) was performed with primer pair PNV7 (5´-AGGTGGTGGCCGAGGAYCTCCT-3´) and PNV8 (5´-TCACCATAGAAGGARAAGCA-3´) targeting the RdRp (211 bp) of QW101 strain.

RT-PCR products were purified with the QIAquick Gel Extraction kit (Qiagen) before cloning into pCR2.1-TOPO (T/A) or PCR XL cloning kit (Invitrogen). Five clones of each sample were sequenced. DNA sequencing was performed with BigDye Terminator Cycle and 3730 DNA Analyzer (Applied Biosystems, Foster City, CA, USA).

Sequence editing was performed by Lasergene software package (v5, DNASTAR Inc., Madison, WI, USA). The Basic Local Alignment Search Tool (BLAST, http://www.ncbi.nlm.nih.gov/BLAST) was used to find homologous hits. Multiple sequence alignment was performed with ClustalW (v1.83) at DNA Data Bank of Japan (http://www.ddbj.nig.ac.jp). Phylogenetic and bootstrap (1,000 replicates) analyses were conducted by using MEGA (v2.1) (*18*). Identification of recombinants was performed by using the Recombinant Identification Program (RIP, http://hivweb.lanl.gov/RIP/RIPsubmit.html) (*19*). The classification and GenBank accession numbers of NoVs are listed in Table 1.

**Table 1:** Classification and GenBank accession numbers of norovirus (NoV) strains used for sequence analysis*

Strain	Genus/genogroup-genotype	Abbreviation	GenBank accession no.
Hu/Norwalk/68/US	NoV/GI-1	Norwalk	M87661
Hu/Hawaii/71/US	NoV/GII-1	Hawaii	U07611
Hu/Melksham/89/UK	NoV/GII-2	Melksham	X81879
Hu/Snow Mountain/76/US	NoV/GII-2†	Snow Mountain	AY134748
Hu/Mexico/89/MX	NoV/GII-3	Mexico	U22498
Hu/Toronto/91/CA	NoV/GII-3	Toronto	U02030
Hu/SaitamaU18/97-99/JP	NoV/GII-3	SaitamaU18	AB039781
Hu/SaitamaU201/98/JP	NoV/GII-3	SaitamaU201	AB039782
Hu/Arg320/ARG	NoV/GII-3†	Arg320	AF190817
Hu/Camberwell/101922/94/AUS	NoV/GII-4	Camberwell	AF145896
Hu/Lordsdale/93/UK	NoV/GII-4	Lordsdale	X86557
Hu/Bristol/93/UK	NoV/GII-4	Bristol	X76716
Hu/MD145-12/87/US	NoV/GII-4	MD145	AY032605
Hu/Farmington Hills/02/US	NoV/GII-4	Farmington Hills	AY502023
Hu/Langen1061/02/DE	NoV/GII-4	Langen	AY485642
Hu/Hillingdon/93/UK	NoV/GII-5	Hillingdon	AJ277607
Hu/New Orleans 306/94/US	NoV/GII-5	New Orleans	AF414422
Hu/Baltimore/274/1993/US	NoV/GII-6	Baltimore	AF414408
Hu/SaitamaU3/97/JP	NoV/GII-6	SaitamaU3	AB039776
Hu/SaitamaU4/97/JP	NoV/GII-6	SaitamaU4	AB039777
Hu/SaitamaU16/97/JP	NoV/GII-6	SaitamaU16	AB039778
Hu/SaitamaU17/97/JP	NoV/GII-6	SaitamaU17	AB039779
Hu/Seacroft/90/UK	NoV/GII-6†	Seacroft	AJ277620
Hu/Leeds/90/UK	NoV/GII-7	Leeds	AJ277608
Hu/Gwynedd/273/94/US	NoV/GII-7	Gwynedd	AF414409
Hu/Amsterdam/98-18/98/NET	NoV/GII-8	Amsterdam	AF195848
Hu/SaitamaU25/97-99/JP	NoV/GII-8	SaitamaU25	AB039780
Hu/VA97207/97/US	NoV/GII-9‡	VA97207	AY038599
Hu/NLV/Erfurt/546/00/DE	NoV/GII-10	Erfurt	AF427118
Hu/Mc37/00-01/THA	NoV/GII-10†	Mc37	AY237415
Po/Sw43/97/JP	NoV/GII-11	Sw43	AB074892
Po/Sw918/97/JP	NoV/GII-11	Sw918	AB074893
**Po/MI-QW48/02/US**	**NoV/GII-11**	**QW48**	**AY823303**
Hu/Gifu/96/JP	NoV/GII-12‡	Gifu	AB045603
HU/Wortley/90/UK	NoV/GII-12†	Wortley	AJ277618
Hu/SaitamaU1/97-99/JP	NoV/GII-12†	SaitamaU1	AB039775
Hu/Fayetteville/98/US	NoV/GII-13	Fayetteville	AY113106
Hu/M7/99/US	NoV/GII-14	M7	AY130761
Hu/J23/99/US	NoV/GII-15	J23	AY130762
Hu/Tiffin/99/US	NoV/GII-16	Tiffin	AY502010
Hu/Neustrelitz260/00/DE	NoV/GII-16	Neustrelitz	AY772730
Hu/CS-E1/02/US	NoV/GII-17	CS-E1	AY502009
**Po/OH-QW101/03/US**	**NoV/GII-18**	**QW101**	**AY823304**
**Po/OH-QW125/03/US**	**NoV/GII-18**	**QW125**	**AY823305**
**Po/OH-QW170/03/US**	**NoV/GII-19‡**	**QW170**	**AY823306**
**Po/OH-QW218/03/US**	**NoV/GII-19‡**	**QW218**	**AY823307**
Bo/Newbury-2/76/UK	NoV/GIII-2	Newbury-2	AF097917
Hu/Alphatron/98-2/98/NET	NoV/GIV	Alphatron	AF195847
Mu/MNV-1/03/US	NoV/GV	MNV-1	AY228235

*Classification is based on the capsid gene sequences. The 5 porcine NoV strains sequenced in this study are in **boldface**.

†Previously reported recombinants (*20**–**24*).

‡Potential recombinants found in this study.

Four gnotobiotic pigs were maintained and euthanized as previously described (*25**,**26*). The inoculate was a 20% fecal filtrate (0.2 μm) in EMEM of the QW126 or QW144 (QW101-like, GII-18) strains or EMEM only (2 negative control pigs). One pig was inoculated with QW126 orally and intranasally at 9 days of age, and convalescent-phase antiserum LL616 was collected at postinoculation day (PID) 26. A second pig was inoculated with QW144 orally at 35 days of age and euthanized at PID 5.

Immune electron microscopy (IEM) was performed as described previously (*27*). For enzyme-linked immunosorbent assay (ELISA), the recombinant baculovirus-expressed human NoV VLPs and rotavirus VP2 and VP6 (2/6)-VLPs (negative control) (*28*) were CsCl-gradients purified. We coated 96-well microplates with VLPs (200 ng/well) in carbonate buffer (pH 9.6) and blocked with 5% nonfat dry milk in phosphate-buffered saline (PBS)-Tween 20 (0.05%). Serially diluted serum samples that included positive and negative controls were added to duplicate positive- and negative-coated wells, and the plates were incubated. After washing, horseradish peroxidase (HRP)-labeled goat anti-pig immunoglobulin G (IgG) (H + L) for pig sera or goat anti-human IgG + IgA + IgM (H + L) (KPL, Gaithersburg, MD, USA) for human serum was added. After incubation and washing, the substrate 3,3´,5,5´-tetramethylbenzidine was added. The cutoff value was the mean absorbance of the negative coatings multiplied by 2.

Western blot was performed as described previously (*29*). Nitrocellulose membranes were incubated with pig convalescent-phase antiserum LL616 against porcine GII-18 NoV or negative control serum in PBS containing 4% nonfat dry milk followed by goat anti-pig IgG (H + L)-HRP conjugate.

## Results

Porcine NoVs were classified into 3 genotypes within GII based on the complete capsid sequences: 1 genotype with prototype Japanese strains Sw43 and Sw918 and 2 new genotypes. A total of 19 of 275 samples showed a potential positive band after agarose gel electrophoresis of the RT-PCR products of primer pair p290/110. Fourteen samples representative of each potentially positive farm or the slaughterhouse were sequenced. After performing BLAST search, we identified 6 NoVs (QW48, Michigan farm A; QW101, QW125, and QW126, Ohio farm B; and QW170 and QW218, Ohio slaughterhouse), 3 sapoviruses, and 5 sequences that had no significant hit in the database. Because the QW126 shared 99% nucleotide (nt) identity with the QW101 and QW125 strains in the 274-nt RdRp region, it was not sequenced further.

We sequenced the 3-kb 3´ end of the genome containing the partial RdRp, VP1 and VP2 genes, and the 3´ untranslated region of the 5 strains. The porcine NoVs represented 3 distinct clusters: 1) Sw43, Sw918, and QW48; 2) QW101 and QW125; and 3) QW170 and QW218, on the basis of the size of each gene and the ORF1-ORF2 overlap region (Table 2). Across the 3 kb, the QW101 and QW125 strains and the QW170 and QW218 strains shared 99% nt identity.

**Table 2:** Sizes of the putative capsid protein VP1 and the minor capsid protein VP2, the overlap regions, and the 3´ UTR of GII NoV*

Species/genogroup-genotype/strain	ORF1-ORF2 overlap (nt)	VP1 (aa)	ORF2-ORF3 overlap (nt)	VP2 (aa)	3´ UTR (nt)
Po/GII-11/Sw43	17	547	NA	NA	NA
Po/GII-11/Sw918	17	547	NA	NA	NA
Po/GII-11/QW48	17	547	1	253	57
Po/GII-18/QW101	20	557	1	275	48
Po/GII-18/QW125	20	557	1	275	48
Po/GII-19/QW170	17	548	1	254	51
Po/GII-19/QW218	17	548	1	254	51
Hu/GII-1/Hawaii	20	535	1	259	42
Hu/GII-2/Snow Mountain	20	542	1	259	45
Hu/GII-3/SaitamaU18	20	548	1	254	37
Hu/GII-4/MD145	20	539	1	268	46
Hu/GII-5/New Orleans	20	540	1	258	35
Hu/GII-6/SaitamaU3	20	550	1	259	54
Hu/GII-7/Gwynedd	20	540	1	257	68
Hu/GII-8/SaitamaU25	20	537	1	257	53
Hu/GII-9/VA97207	20	537	1	257	51
Hu/GII-10/Mc37	20	548	1	258	34
Hu/GII-12/SatamaU1	20	535	1	259	50
Hu/GI-1/Norwalk	17	530	1	212	66

*UTR, untranslated region; NoV, norovirus; ORF, open reading frame; nt, nucleotide; aa, amino acid; NA, not available.

**Figure 1:**
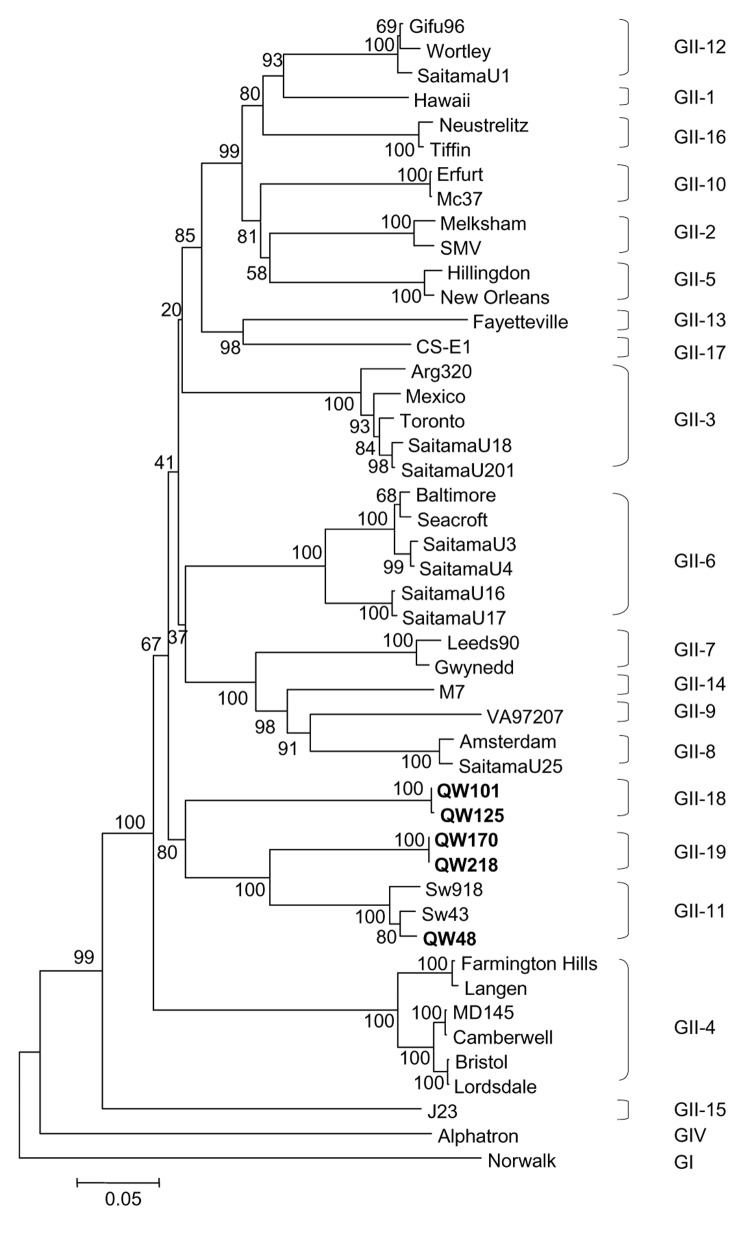
Neighbor-joining phylogenetic tree of genogroup II noroviruses (NoVs) based on the complete capsid region. The 5 newly identified porcine NoV strains are in boldface. Genogroups (G) and genotypes (numbers after G) are indicated. The human NoV GI-1/Norwalk and GIV/Alphatron strains were used as outgroup controls.

The amino acid identity of the predicted complete and S and P domains of the capsid protein of the 5 porcine NoVs, the previously reported porcine NoVs (Sw43 and Sw918), and representative human, bovine, and murine NoV strains is summarized in Table 3. In the complete capsid, the QW48 strain was most closely related to the porcine NoV prototype Sw43 strain (98% amino acid identity); the QW170 and QW218 strains shared the highest amino acid identities (81%) to porcine Sw43 and Sw918 strains; the QW101 and QW125 strains showed the highest amino acid identity to human GII-3/Mexico (71.4%), then to human GII-6/Baltimore (71.0%), porcine QW218 (71.0%), and porcine Sw43 (70.6%) strains. The S and P domains of these NoVs showed similar relationships. A neighbor-joining phylogenetic tree based on the amino acid sequences of the complete capsids (Figure 1) showed that QW48 grouped with Sw43 and Sw918 strains into GII-11 and that QW170 and QW218 formed a new genotype (GII-19), which was closer to porcine than to human strains. However, QW101 and 125 formed a new genotype (GII-18) between human and porcine GII NoVs.

**Table 3:** Percentage amino acid identities of noroviruses within the capsid region

Strain	Complete capsid (S domain, P domain)					
	Po/GII*	Hu/GII†	Hu/GI/Norwalk	Bo/GIII/Newbury-2	Hu/GIV/Alphatron	Mu/GV/MNV-1
QW48	96–98 (100, 94–97)	63–71 (77–85, 53–63)	43 (59, 36)	45 (62, 36)	53 (71, 42)	39 (58, 29)
QW101, QW125	70–70.6 (83, 63)	61–71.4 (77–86, 51–64)	42 (59, 35)	45 (62, 38)	54 (71, 44)	39 (58, 28)
QW170, QW218	81 (90, 74)	62–69 (77–82, 52–62)	43 (59, 36)	45 (61, 37)	53 (72, 40)	39 (60, 27)

*Includes Sw43 and Sw918 strains.

†Includes Hawaii, Snow Mountain, Mexico, MD145, New Orleans, Baltimore, Gwynedd, Amsterdam, VA97207, Erfurt, Gifu, Fayetteville, M7, J23, and Neustrelitz strains.

**Figure 2:**
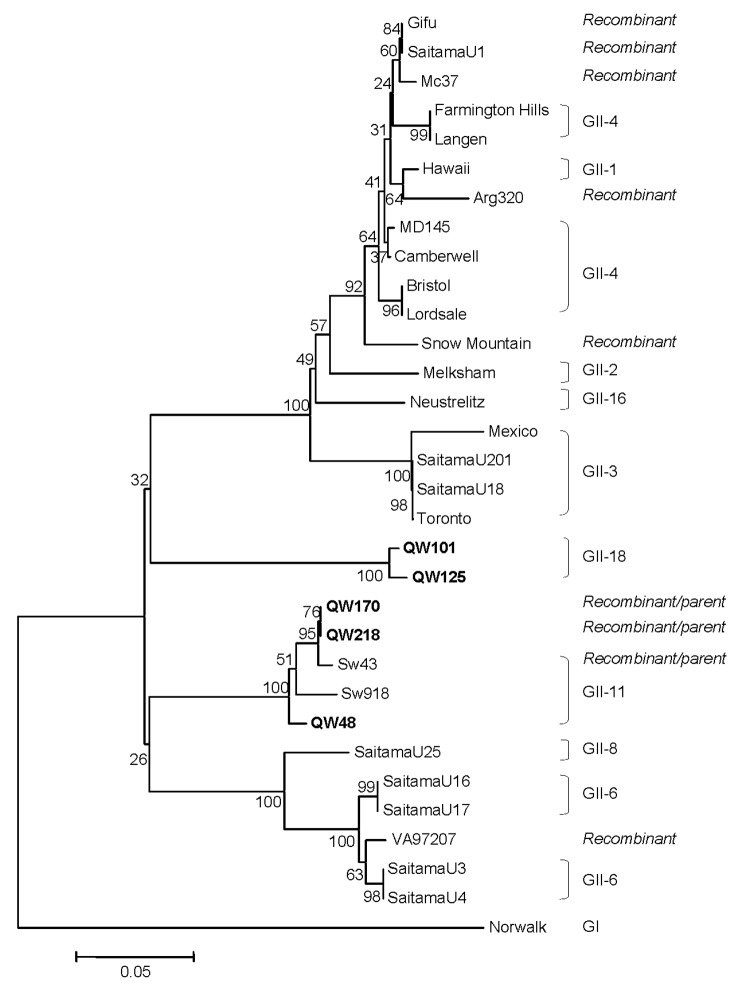
Neighbor-joining phylogenetic tree of genogroup II noroviruses (NoVs) based on the partial RNA-dependent RNA polymerase region (C-terminal 260–266 amino acids). The 5 newly identified porcine NoV strains are in boldface. Genogroups (G) and genotypes (numbers after G) are indicated. The human NoV GI-1/Norwalk strain was used as outgroup control.

Further analysis of the predicted C-terminal ≈260 amino acids of the RdRp region (Figure 2) showed similar grouping results for QW48, QW101, and QW125 strains but different for QW170 and QW218 strains, which were in the same cluster (GII-11) as Sw43, Sw918, and QW48 in the RdRp region. This finding suggested that a recombination event occurred between QW170/218-like and Sw43-like NoVs. The complete VP2 sequences of representative strains were also analyzed (data not shown). Results were similar to those of the capsid sequence classification.

**Figure 3:**
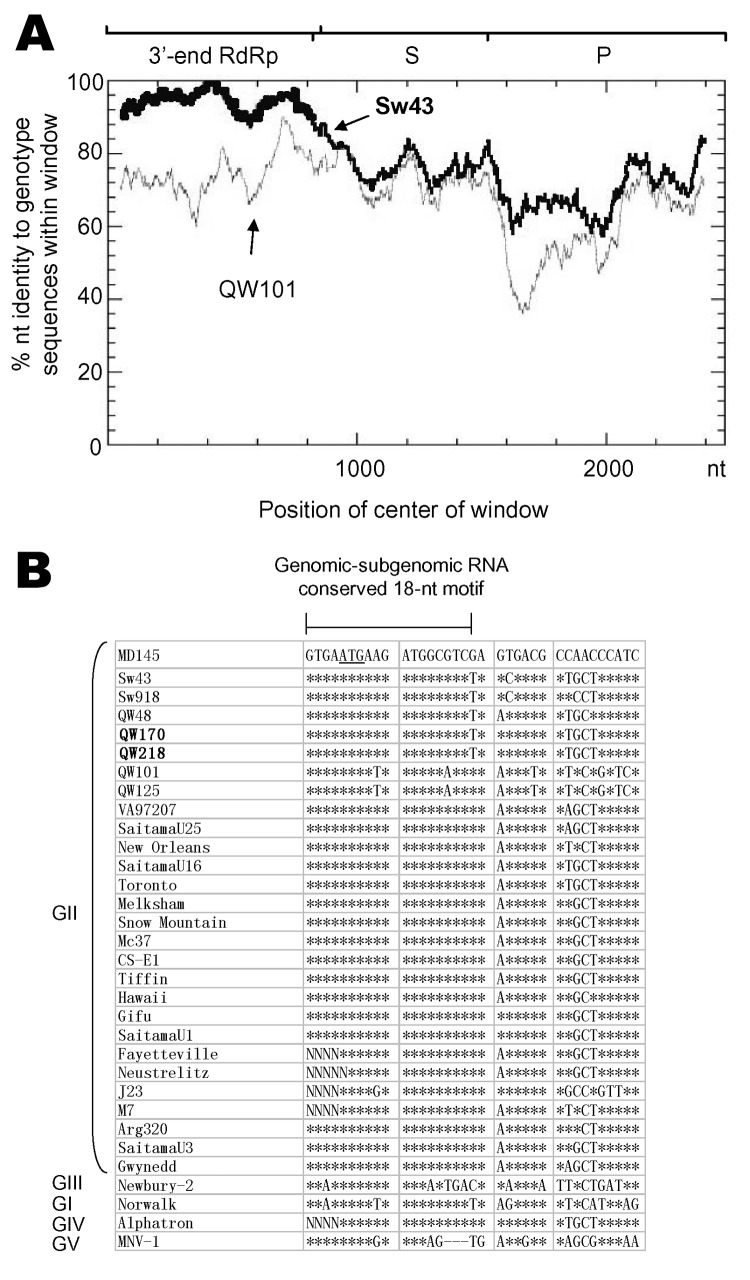
Identification of a potential recombination event between QW170 and Sw43 strains. A) Recombination Identification Program analysis of QW170 strain. At each position of the window, the query sequence (QW170) was compared to each of the background genotype representatives (GII-11/Sw43 and GII-18/QW101). When the query sequence is similar to the background sequences, the homologous regions are indicated as thick lines on the plot. Analysis parameters were window size of 100 and significance of 90%. The nucleotide positions of the 3´-end RNA-dependent RNA polymerase (RdRp) and the shell (S) and protruding (P) domains of the capsid protein are indicated. B) Sequence alignments of the RdRp-capsid junction region of noroviruses (NoVs). The genomic and subgenomic conserved 18-nucleotide (nt) motif is indicated by a horizontal line with 2 vertical bars. Asterisks indicate the identical residues to the sequence of the first line. Dashes represent gaps. The letter N indicates missing data on the residue. The start codon of open reading frame ORF 2 is underlined. Five NoV genogroups are indicated.

A potential recombination event occurred between QW170/218-like and Sw43-like strains. To examine where the recombination occurred, we performed RIP analysis by placing the 3´-end RdRp and the capsid sequence of QW170 or QW218 as a query sequence and the corresponding sequences of Sw43 and QW101 as background sequences. The resulting diagram (Figure 3A) showed that QW170 had high similarity to Sw43 in the RdRp but not in the capsid region. This abrupt change happened in the RdRp-capsid junction region. Therefore, we performed sequence alignments of the RdRp-capsid junction of NoVs, including the calicivirus genomic-subgenomic conserved 18-nt motif (*20*) (Figure 3B). Between Sw43, QW170, and QW218, all 18 nt were identical, but identities decreased downstream of this motif. QW170 and QW218 grouped with Sw43 with a high bootstrap value of 95 in the RdRp tree (Figure 2), whereas they segregated from Sw43 with the highest bootstrap value of 100 in the capsid tree (Figure 1). We could not clarify which was the parent or progeny strain.

**Figure 4:**
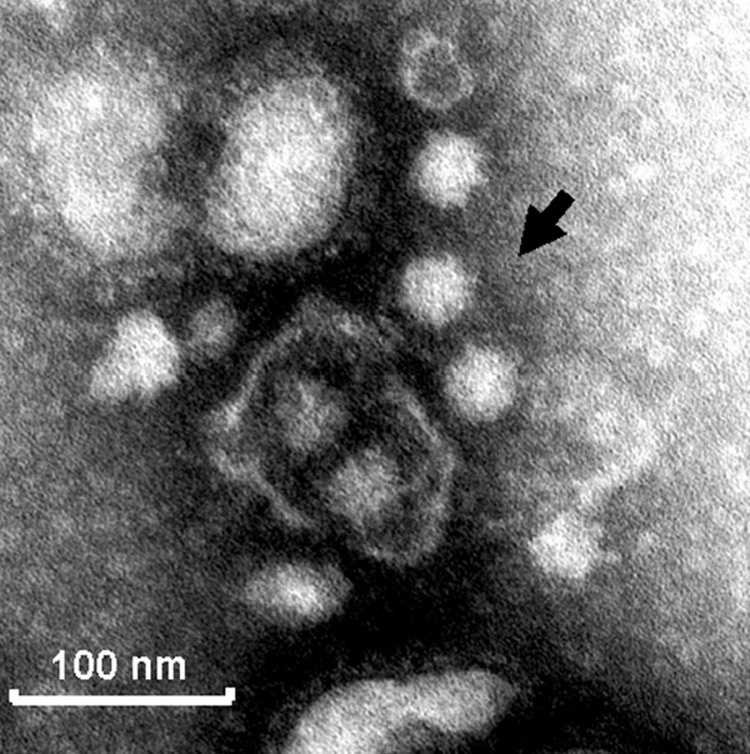
Immune electron micrograph of porcine noroviruses (NoVs). The diluted intestinal contents of a gnotobiotic pig euthanized on postinoculation day 5 to QW101-like porcine NoVs (QW144) were incubated with convalescent-phase serum LL616 from another gnotobiotic pig inoculated with QW101-like porcine NoVs (QW126) and visualized by negative staining with 3% phosphotungstic acid. The arrow indicates a small clump of NoV-like particles.

The porcine NoVs replicated in gnotobiotic pigs. Two pigs were inoculated with QW101-like GII-18 porcine NoVs (QW126 and QW144 strains) to verify their replication in pigs as confirmed by quantitative RT-PCR and IEM and to produce convalescent-phase serum to examine antigenic reactivity with human NoVs. These 2 strains were confirmed as QW101-like porcine NoVs in both the RdRp (169-nt) and the capsid S domain (363-nt) regions by sequence analysis of the RT-PCR products (Q.H. Wang and L.J. Saif, unpub. data). They shared 99% and 100% amino acid identities to the QW101 strain in the 2 regions, respectively. Porcine NoV shedding, assessed by quantitative RT-PCR with primer pair PNV7/8, was detected at PID 3–5 (euthanized) after QW144 exposure, coincident with mild diarrhea. The RT-PCR–detectable units of the rectal swab RNA increased from negative at PID <2, 10^3^ at PID 3–4, and 10^4^ at PID 5 (large intestinal contents). Norovirus shedding was detected only at PID 5 without diarrhea after QW126 exposure. Examination of the intestinal contents of the pig inoculated with QW144 by IEM with pig convalescent-phase antiserum LL616 showed clumps of ≈32-nm NoV particles (Figure 4). The 2 control pigs had no virus shedding or diarrhea. Detailed studies of the pathogenesis of porcine NoVs in gnotobiotic pigs are in progress (S. Cheetham and L.J. Saif, unpub. data).

**Figure 5:**
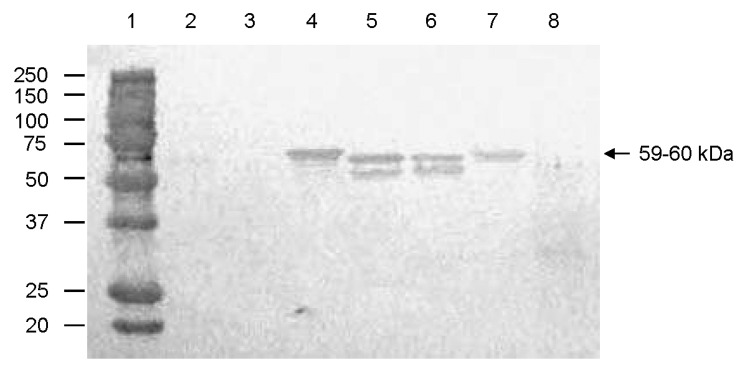
Antigenic cross-reactivity between human genogroup (G) II norovirus (NoV) capsid proteins and a pig convalescent-phase antiserum (LL616) against porcine QW101-like (GII-18) NoV was determined by Western blot. The CsCl-gradient purified viruslike particles (1,250 ng) were separated by sodium dodecyl sulfate 10% polyacrylamide gel electrophoresis, blotted onto nitrocellulose membranes, and tested with LL616. The sucrose-cushion (40%, wt/vol) purified Sf9 insect cell proteins acted as a negative control (lane 8). Lane 1, molecular weight marker (kDa); lanes 2–7, Hu/GI-3/Desert Shield, Hu/GII-1/Hawaii, Hu/GII-3/Toronto, Hu/GII-4/MD145, Hu/GII-4/HS66, and Hu/GII-6/Florida, respectively.

Antisera to QW101-like (QW126) porcine NoVs cross-reacted with VLPs of human GII NoVs in ELISA and Western blot. In ELISA (Table 4), the pig convalescent-phase antiserum (LL616) to QW101-like porcine NoV QW126 strain showed higher titers (1:400–1:800) to GII-3/Toronto, GII-4/MD145, GII-4/HS66, and GII-6/Florida strains; a lower titer (1:100) to GII-1/Hawaii strain; and lowest titer (1:10) to GI-3/Desert Shield strain. In Western blot (Figure 5), the capsid proteins (59–60 kDa) of Toronto, MD145, HS66, and Florida strains, but not the Hawaii and Desert Shield strains, were detected by pig antiserum LL616 but not the negative control serum (data not shown). Thus, 1-way antigenic cross-reactivity exists between human NoV antigens and porcine NoV (GII-18) antiserum, with moderate cross-reactivity to human NoVs GII-3, 4, and 6; low cross-reactivity to GII-1; and very low cross-reactivity to GI-3.

**Table 4:** Antigenic cross-reactivity between human GII NoV antigens (VLPs) and a pig convalescent-phase antiserum against porcine GII NoVs, as determined by ELISA*

Antiserum	ELISA antibody titer with each VLP antigen (genogroup-genotype)					
	Hawaii (GII-1)	Toronto (GII-3)	MD145 (GII-4)	HS66 (GII-4)	Florida (GII-6)	Desert Shield (GI-3)
HS66CS (positive control): human convalescent antiserum to human HS66 (GII-4)	1:25,600	1:6,400	1:25,600	1:25,600	1:6,400	1:6,400
LL616: pig convalescent-phase antiserum to porcine QW126 (QW101-like, GII-18)†	1:100	1:800	1:400	1:400	1:400	1:10
LL368 (negative control): preinoculation serum‡	<1:10	<1:10	<1:10	<1:10	<1:10	<1:10
MM982 (negative control): preinoculation serum‡	<1:10	<1:10	<1:10	<1:10	<1:10	<1:10

*NoV, norovirus; VLP, viruslike particle; ELISA, enzyme-linked immunosorbent assay.

†The QW126 shared 99% and 100% amino acid identities to the QW101 strain (GII-18) for a 169-bp segment in the RNA-dependent RNA polymerase region and a 363-bp segment in the capsid region, respectively.

‡LL368 and MM982 were sera from 2 gnotobiotic pigs before inoculation with porcine NoVs.

## Discussion

All porcine NoVs were detected from pigs without clinical signs (*9**,**10*). Subclinically infected pigs may be natural reservoirs for NoVs, and because porcine GII NoVs are genetically and antigenically related to human NoVs, concerns exist about their zoonotic potential. Whether human NoV strains similar to the QW101-like porcine NoVs circulate among people with occupational exposure to pigs is unknown, but such studies could provide information on the zoonotic potential of these porcine NoVs.

The RdRp-capsid junction region of NoVs contains a highly conserved 18-nt motif in genomic and subgenomic RNA that is believed to be a transcription start signal (*1**,**20*). All 18 nt were identical within each genogroup except for the Hu/GII/J23, Po/GII/QW101, and Po/GII/QW125 strains (Figure 3B, sequence alignments on other GI and GIII strains are not shown). This finding suggests that homologous recombination may occur within this motif between NoVs of different genotypes within the same genogroup. Recombinant human GII NoVs have been reported previously (*20**–**24*). To our knowledge, this study is the first identification of a potential recombinant between pig NoVs. At present, NoV recombinants have been detected exclusively between viruses within the same genogroup and within the same host species, but few animal NoVs have been sequenced (RdRp and capsid) for comparative analysis, especially those from animals in developing countries, where humans and animals may be in close contact.

The QW101-like porcine NoVs replicated in gnotobiotic pigs with fecal shedding, documented by quantitative RT-PCR and IEM. No cell culture system or animal disease models are available for human NoVs, which impedes the study of their pathogenesis, replication strategies, host immune responses, and preventive approaches. The infection of pigs with porcine NoVs may provide a new infection or disease model to study NoV infections.

In this study, 1-way antigenic cross-reactivity occurred between antiserum to QW101-like porcine NoVs and the capsid proteins of human NoVs, with highest cross-reactivity to GII-3, 4, and 6 NoVs. This finding coincides with the finding that the QW101 strain shares high amino acid identity with GII-3 (71%), GII-6 (71%), and GII-4 (63%) NoVs.

In summary, 3 genotypes of porcine NoVs were detected in US swine. One genotype (QW101-like, GII-18) was genetically and antigenically most closely related to human GII NoVs. Potential recombinant porcine NoV strains were identified. The QW101-like NoVs infected gnotobiotic pigs, and NoV particles were evident in intestinal contents. These results raise questions of whether pigs may be reservoirs for emergence of new human NoVs or if porcine/human GII recombinants could emerge.
